# Dysfunction of CCR1^+^ decidual macrophages is a potential risk factor in the occurrence of unexplained recurrent pregnancy loss

**DOI:** 10.3389/fimmu.2022.1045532

**Published:** 2022-12-02

**Authors:** Yifei Sang, Yanhong Li, Ling Xu, Jiajia Chen, Dajin Li, Meirong Du

**Affiliations:** ^1^ National Health Council (NHC) Key Laboratory of Reproduction Regulation, Shanghai Institute of Planned Parenthood Research, Obstetrics and Gynecology Hospital, Fudan University, Shanghai, China; ^2^ Shanghai Key Laboratory of Female Reproductive Endocrine Related Diseases, Obstetrics and Gynecology Hospital, Fudan University Shanghai Medical College, Shanghai, China; ^3^ Department of Obstetrics and Gynecology, Shanghai Fourth People’s Hospital, School of Medicine, Tongji University, Shanghai, China; ^4^ State Key Laboratory of Quality Research in Chinese Medicine and School of Pharmacy, Macau University of Science and Technology, Macau, Macau SAR, China

**Keywords:** decidual macrophages, CCR1, CCL8, trophoblasts, epithelial-to-mesenchymal transition, unexplained recurrent pregnancy loss

## Abstract

Recurrent pregnancy loss (RPL) puzzles 1–3% of women of childbearing age worldwide. Immunological factors account for more than 60% of cases of unexplained RPL (URPL); however, the underlying mechanism remains unclear. Here, using single-cell sequencing data and functional experiments with clinical samples, we identified a distinct population of CCR1^+^ decidual macrophages (dMφ) that were preferentially enriched in the decidua from normal early pregnancies but were substantially decreased in patients with URPL. Specific gene signatures endowed CCR1^+^ dMφ with immunosuppressive and migration-regulatory properties, which were attenuated in URPL. Additionally, CCR1^+^ dMφ promoted epithelial-to-mesenchymal transition (EMT) to promote trophoblast migration and invasion by activating the ERK1/2 signaling pathway. Decidual stromal cell (DSC)-derived CCL8 was the key regulator of CCR1^+^ dMφ as CCL8 recruited peripheral CCR1^+^ monocytes, induced a CCR1^+^ dMφ-like phenotype, and reinforced the CCR1^+^ dMφ-exerted modulation of trophoblasts. In patients with URPL, CCL8 expression in DSCs was decreased and trophoblast EMT was defective. Our findings revealed that CCR1^+^ dMφ play an important role in immune tolerance and trophoblast functions at the maternal–fetal interface. Additionally, decreased quantity and dysregulated function of CCR1^+^ dMφ result in URPL. In conclusion, we provide insights into the crosstalk between CCR1^+^ dMφ, trophoblasts, and DSCs at the maternal–fetal interface and macrophage-targeted interventions of URPL.

## Introduction

The development of the semi-allogeneic fetus without rejection by the maternal immune system has aroused widespread concern in reproductive immunology. Delicate maternal immunomodulation depends on the crosstalk between decidual immune cells (DICs), decidual stromal cells (DSCs), and fetal-derived trophoblast cells, which play key roles in embryo implantation, maternal–fetal immune tolerance, placenta formation, and spiral artery remodeling (1–4). Dysregulated immunology disturbs the unique maternal–fetal immune environment, causing deficient decidualization, embryo rejection, and placental malformation, which eventually result in various pregnancy complications, including unexplained recurrent pregnancy loss (URPL) (5–7). Recurrent pregnancy loss (RPL), defined as two or more pregnancy loss before 20-24 weeks of gestation, is a devastating health problem that affects 1–3% of reproductive women (8). In approximately 50% of these patients, RPL has an unknown etiology, defined as URPL (8). Immunological factors account for more than 60% of URPL cases (9). However, the underlying mechanism remains unclear.

As the second-most abundant immune cell at the maternal–fetal interface, decidual macrophages (dMφ) are multifunctional and actively participate in the establishment of a tolerogenic immune microenvironment, defense from invaders, tissue repair and remodeling, scavenging apoptotic cells, and regulation of trophoblast cell activity (10–12). Evidence from studies suggests that dMφ are enriched in the vicinity of the trophoblast invasion front and promote trophoblast migration and invasion, as well as the replacement of vascular smooth muscle cells in spiral artery remodeling (13, 14). However, dMφ from women with URPL show M1-like features with increased expression of activation molecules (such as CD80 and CD86) and inflammatory cytokines but reduced production of anti-inflammatory cytokines such as IL-10 (15). This transformation of dMφ in URPL from an immunosuppressive M2-like phenotype to a pro-inflammatory M1-like phenotype results in adverse effects on the migratory and invasive activity of trophoblast cells (16). Our knowledge of dMφ is still limited to the M1-M2 classification (17, 18), which may not be adequate for understanding their precise roles and changes in the complicated pathological context of URPL. Whether a specific dMφ subset plays a role in the occurrence of URPL? What are the regulatory factors of the subset and mechanisms by which they mediate the phenotypic and functional changes in these cells in URPL? These are the main subjects that we try to explore in this study.

CCR1, as a G protein–coupled receptor for a C-C type chemokine that is broadly expressed by various cells, including tumor cells, myeloid-derived suppressor cells, and monocytes/macrophages (19–21). CCR1, with the highest ligand promiscuity, is a crucial component in cell migration, cell differentiation, immune responses, immune regulation, and other pathophysiological processes by binding to its relative ligands, including CCL3, CCL5-9 (CCL6 and CCL9 are of mouse origin), CCL13-16 and CCL23 (22–24). Previous studies have demonstrated that CCR1 mediates the migration and recruitment of peripheral monocytes and accumulation of metastasis-associated macrophages (25, 26). Moreover, recruitment of CCR1-expressing myeloid cells promotes tumor invasion and metastasis in colorectal cancer (27). In addition, single-cell analysis revealed that CCL3–CCR1 interactions increased macrophage recruitment and anti-inflammatory patterns in tumors (20). Notably, CCR1 expression is more abundant in M2 Mφ than in M1 Mφ (28). Deletion of CCR1 results in fewer M2 Mφ during mammary gland development (29). CCL5 also directly motivates M1 polarization and inhibits M2 polarization through CCR1-mediated activation of the MAPK and NF-κB pathways in drug-induced liver injury (30). Therefore, functional regulation of CCR1 on Mφ is tissue- and ligand-specific. Few studies have reported on CCR1 in pregnancy, except for CCR1 expression in leukocytes, DSCs, glandular epithelium, and luminal epithelium by immunohistochemical staining of the decidual tissue (31). CCR1-mediated modulation on dMφ implicated in normal pregnancy and URPL remains undisclosed and is our concern.

In this study, we aimed to analyze CCR1 expression in dMφ of women with URPL and normal pregnancy and investigate the phenotype and function of CCR1^+^ dMφ in the patient and control groups. Additionally, we assessed the regulation of CCR1^+^ dMφ by DSC-derived CCL8. Our data demonstrate that CCR1^+^ dMφ are preferentially enriched in the decidua from normal early pregnancies and significantly decreased in patients with URPL. More importantly, CCR1^+^ dMφ in URPL displayed distinct transcriptional profiles and functions compared with those in normal controls. This unique dMφ subset, modulated by CCL8, contributed to the maternal-fetal immune tolerance and the normal function of trophoblast cells. In summary, we provide insights into maternal–fetal crosstalk and suggest CCR1^+^ dMφ or CCL8/CCR1 signaling as a potential target to prevent URPL.

## Materials and methods

### Subjects and human sample collection

The collection and use of human tissue samples were approved by the Human Research Ethics Committee of the Obstetrics and Gynecology Hospital, Fudan University, and followed the principles of the Helsinki Declaration (0423-10-HMO). Written informed consent was obtained from all women in this study. Endometrial tissues (n=20) were collected from patients with leiomyomas during hysterectomy. Patients aged between 25 and 40 years with regular menstrual cycles who had not received hormone therapy or took any medications were considered for this study. Normal decidua, villous tissues, and peripheral blood were obtained from women with clinically normal pregnancies (NPs; terminated for nonmedical reasons, gestational age: 6-10 weeks). All women in this study who had NPs never had spontaneous miscarriage and had at least one live birth before the current pregnancy. Decidual tissues and peripheral blood were collected from patients with URPL who received uterine curettage because of a lack of fetal heartbeat detected by ultrasound at 6-10 weeks of gestation. All URPL subjects had regular ovulatory menstrual cycles and a history of two or more consecutive miscarriages, excluding genetic, anatomic, or endocrine abnormalities; infections; immune disorders (anti-phospholipid antibody syndrome, thrombophilia, systemic lupus erythematosus, Hashimoto’s thyroiditis, Graves’ disease, rheumatoid arthritis); or poor health habits. Demographic details and characteristics of women with NPs (n=69) and URPL (n=25) are shown in **Table 1**. All samples were obtained under sterile conditions and divided into two parts: One part was immediately fixed in 4% paraformaldehyde for immunohistochemistry (IHC) studies, and the other part was immediately collected into ice-cold DMEM/F12 medium (Gibco, USA), transported to the laboratory within 30 min, and washed in 1× sterile phosphate buffered saline (PBS) for cell isolation.

**Table 1:** Demographics and clinical characteristics of the population.

	NP (n=69)	URPL (n=25)
Age (years)	29.36 ± 0.50	30.68 ± 0.71
Gestational age (weeks)	8.02 ± 0.11	8.28 ± 0.18
Gravidity	2.52 ± 0.08	3.6 ± 0.14****
Parity	1.2 ± 0.05	0.12 ± 0.07****
Number of abortions	0.32 ± 0.06	2.48 ± 0.13****
Number of live births	1.21 ± 0.05	0.12 ± 0.07****

****p < 0.0001.

### Cell lines

HTR-8 cells, an immortalized first-trimester trophoblast cell line, were purchased from the Cell Bank of the Chinese Academy of Sciences, Shanghai, China, and cultured in DMEM/F12 medium (Gibco, USA) supplemented with 10% fetal bovine serum (FBS) (Gibco, USA).

### Isolation and culture of human endometrial stromal cells (ESCs)

Endometrium tissues were washed twice in 1× PBS and cut and digested in DMEM/F12 medium supplemented with 1.0 mg/ml type IV collagenase (Sigma-Aldrich, USA) for 30 min at 37°C with gentle agitation. The suspension was filtered through a 40-μm nylon mesh (Falcon, USA) and centrifuged at 300×g for 8 min. The collected cells were cultured in phenol red-free DMEM/F12 medium (Genom, China) containing 10% charcoal-stripped FBS (BioSun, China), 1% ITS (Oricellbio, China), and 500 ng/mL puromycin for 24 h in a 37°C humidified incubator containing 5% CO_2_. Adherent cells (ESCs) were digested and resuspended in complete medium for other treatments.

### Isolation and culture of human DSCs and immune cells

Decidual tissues were trimmed into 1-mm^3^ segments and submerged in DMEM/F12 medium supplemented with 1.0 mg/ml type IV collagenase (Sigma-Aldrich, USA) and 150 U/ml DNase I (Sigma-Aldrich, USA). Digestion was performed for 30 min at 37°C with gentle shaking. After digestion, the cells were washed in sterile PBS and filtered through 100, 70, and 40μm sieves. The filtered suspension was centrifuged at 300×g for 8 min and the collected cells were resuspended in DMEM/F12 medium. The suspension was layered on a discontinuous Percoll density gradient (20%/40%/60%; GE Healthcare, USA) and centrifuged for 30 min at 800×g. DSCs were isolated from the 20%/40% Percoll interface, and immune cells were distributed at the 40%/60% Percoll interface. The cells were then washed in sterile PBS for twice. DSCs were cultured in DMEM/F12 medium (Genom, China) containing 10% FBS (Gibco, USA) for 24 h in a 37°C humidified incubator containing 5% CO_2_. Immune cells were collected and resuspended in Roswell Park Memorial Institute (RPMI) 1640 medium supplemented with 10% FBS for further experiments.

### Isolation of CCR1^+^ dMφ

dMφ were isolated from DICs using APC anti-human CD14 (BioLegend, USA) and Anti-APC MultiSort Kit (Miltenyi Biotec, Germany) according to the manufacturer’s protocol. Next, to separate CCR1^-^ and CCR1^+^ dMφ, the magnetic particles were first removed from dMφ by using the MultiSort Release Reagent (Miltenyi Biotec, Germany). Then dMφ were labeled with CCR1-fluorescein isothiocyanate (FITC) antibody (BioLegend, USA) followed by subsequent incubation with anti-FITC microbeads (Miltenyi Biotec, Germany). Then, magnetic separation was applied to positively select CCR1^+^ dMφ subset. The purity of CCR1^+^ dMφ was greater than 90%, as measured by FCM. The cells were cultured in DMEM-F12 medium supplemented with 10% FBS, 100 U/mL penicillin, and 100 mg/mL streptomycin.

### Preparation of peripheral blood mononuclear cells (PBMCs)

PBMCs were isolated from peripheral blood samples of patients with NPs and URPL using Ficoll density gradient centrifugation (Solarbio, China) at 800×g for 20 min. CCR1^+^ peripheral monocytes (pMo) were isolated using the same method as that for CCR1^+^ dMφ. The cells were treated with 50 ng/ml recombinant human macrophage colony-stimulating factor (M-CSF) (MedChemExpress, China).

### Reagents and cell treatments

To induce *in vitro* decidualization, ESCs were plated on tissue culture plates, treated with complete medium containing 0.5 mM 8-bromoadenosine 3′,5′-cyclic monophosphate (8-Br-cAMP) (Sigma-Aldrich, USA) and 100 ng/ml MPA (Sigma-Aldrich, USA), and the medium was given fresh every other day and cells were treated for 4 days. The supernatant of cultured cells was collected and stored at −80°C for further analysis.

PBMCs were treated with 100 ng/ml rhCCL8 (R&D Systems, USA) with or without the administration of CCR1 antagonist (BX471) (MedChemExpress, China) at a concentration of 20 μM. A co-culture system of PBMCs and DSCs with or without the addition of 4 µg/mL CCL8 neutralizing antibody (R&D Systems, USA) was established *via* a 0.4-μm pore size Transwell co-culture system (Corning, USA). The neutralizing antibody was pretreated for 2 h. Briefly, PBMCs were placed in the lower chambers and DSCs were seeded in the upper chambers for 24 h before harvest.

A co-culture system of HTR-8 cells with CCR1^-^ dMφ or CCR1^+^ dMφ pretreated with control medium or recombinant human CCL8 (rhCCL8) (R&D Systems, USA) at concentrations of 10, 50, 100, and 200 ng/ml was established using a Transwell co-culture system (0.4-μm pore size, Corning, USA). Briefly, CCR1^-^ or CCR1^+^ dMφ were placed into the upper chambers and HTR-8 cells were seeded into the lower chambers. The ERK1/2 inhibitor PD98059 (MedChemExpress, China) was used at a concentration of 30 μM.

### Flow cytometry (FCM)

Cells were washed in PBS and incubated with fluorochrome-conjugated antibodies for 30 min at 4°C for cell surface staining. The following specific anti-human monoclonal antibodies were used: PerCP-Cy5.5-conjugated anti-CD45, FITC- or APC-conjugated anti-CD14, PE-Cy7- or FITC-conjugated anti-CCR1, AF700-conjugated anti-CD206, PE-conjugated anti-CD163, anti-CD80, and BV421-conjugated anti-CD86. Cells were fixed and permeabilized with BD Cytofix/Cytoperm™ Fixation/Permeabilization Kit (BD Biosciences, USA) according to the manufacturer’s protocol. The permeabilized cells were stained for intracellular cytokines as follows: APC-conjugated anti-IL-10, anti-TGF-β, and PE-conjugated anti-IL-8. All antibodies were purchased from BioLegend. FCM was performed using CytoFLEX (Beckman Coulter, USA), and the data were analyzed using FlowJo Version 6.1 software (TreeStar, USA).

### Enzyme-linked immunosorbent assay (ELISA)

The secretion of CCL8 in the cultured supernatant samples was determined using ELISA with the human CCL8 ELISA Kit (Abcam, UK) according to the manufacturer’s protocol. Absorbance was measured using a spectrophotometer (Biotek, Vermont, USA) at 450 nm.

### RNA isolation and quantitative real-time polymerase chain reaction

Total RNA was extracted using TRIzol reagent (Invitrogen, USA) and reverse-transcribed into first-strand cDNA (TaKaRa Biotechnology, Japan) according to the manufacturer’s instructions. The synthesized cDNA was amplified using specific primers (Sagon, China) and SYBR Green (Yeasen, China) on the ABI PRISM 7900 Sequence Detection System (Applied Biosystems, USA). The reactions were run in duplicate using RNA samples. Fold change in the expression of each gene was calculated using the 2^−△△CT^ method, with actin as an internal control. According to the existing gene sequences in GenBank, primers were designed using computer assistance, and the primer sequences are shown in **Table 2**.

**Table 2:** Primer sequences.

Gene	Forward sequence	Reverse sequence
CCL8	CAGTTTCCATTCCAATCACCTG	TTGGTGATTCTTGTGTAGCTCT
ACTIN	CATGTACGTTGCTATCCAGGC	CTCCTTAATGTCACGCACGAT

### IHC

IHC was performed on specimens of the human endometrium and decidual tissues from patients with NPs and URPL and villus tissues from patients with NPs. Paraffin-embedded sections were cut to a thickness of 3 µm. Tissue slides were incubated at 60°C for 2 h, and sections were deparaffinized and rehydrated in the order of using xylene and graded ethanol (100%, 95%, 85%, 75%, and 50%). They were soaked in 0.01 M citric acid (pH 6.0) for 20 min at 95°C for antigen retrieval. The slides were then incubated in 3% H_2_O_2_ for 10 min, blocked with 5% BSA for 20 min at room temperature, and incubated with rabbit anti-CCL8 antibody (Abcam, UK) at 4°C overnight in a humidity chamber. The samples were stained with an anti-rabbit IgG secondary antibody for 30 min and incubated in a DAB substrate solution until the desired staining intensity was reached at room temperature. The sections were then counterstained with hematoxylin, washed in running tap water for 30 min, dehydrated, and cleared through a graded series of ethanol (50%,75%, 85%, 95%, and 100%) and xylene. Finally, the sections were sealed with neutral resin and analyzed using an optical microscope (Olympus BX53, Japan).

### Western blot (WB)

Cell lysates were prepared in radioimmunoprecipitation assay lysis buffer (Beyotime, China) containing 1% proteinase inhibitor solution (Beyotime, China) and 1% phosphatase inhibitors (NCM Biotech, China). The protein yield was quantified using a bicinchoninic acid protein assay (Beyotime, China), and the protein was boiled with a 5× loading buffer (NCM Biotech, China). Total protein samples (20 μg) were separated using SDS–PAGE (Beyotime, China) and transferred to polyvinylidene difluoride membranes (Millipore, USA) for 1 h. Nonspecific binding sites were blocked by incubating the membranes with the QuickBlock™ Blocking Buffer (Beyotime, China) for WB for 10 min, followed by overnight incubation with primary antibodies against E-cadherin (1:5000; ProteinTech, USA), N-cadherin (1:2000; ProteinTech, USA), vimentin (1:1,000; PTM bio, China), ERK1/2 (1:1000, Proteintech, USA), p-ERK1/2 (1:1000, ProteinTech, USA), and tubulin (1:1000; Beyotime, China) diluted in blocking buffer (Beyotime, China) overnight at 4°C with gentle shaking. Primary antibodies were removed by washing the membranes three times in TBS-T, and the membranes were incubated for 1 h with secondary antibody (1:5000, arigo Biolaboratories Corp, China) at room temperature. After washing three times with TBS-T, immuno-positive bands on the blots were visualized using an enhanced chemiluminescence detection system (NCM Biotech, China).

### Scratch wound healing assay

Scratch wound healing assay was performed to assess cell motility. HTR-8 cells were seeded in six-well plates. CCR1^-^ and CCR1^+^ dMφ were pretreated with or without 100 ng/ml rhCCL8 for 24 h. When the HTR-8 cell density reached 80%, scratches were made with 1-ml pipette tips, and wounded monolayers were washed three times with PBS, followed by co-culture with pretreated CCR1^-^ and CCR1^+^ dMφ in a 0.4-μm pore size Transwell co-culture system with the addition of serum-free medium. Cells were incubated with 5% CO_2_ at 37°C for 24 h, and the wound healing rates were determined and photographed. The images were analyzed and calculated using ImageJ software (NIH, USA).

### Cell migration assays

An 8-µm pore size filter (Corning, USA) was used to perform Transwell migration. CCR1^+^ pMo and dMφ were seeded into the upper chamber without serum and complete medium with 10% serum with or without 100 ng/ml rhCCL8 was added to the lower chamber. Cells migrated to the lower chamber after 48 h. Cells were gently washed with PBS and fixed with 4% paraformaldehyde and then stained with crystal violet. Images were taken using a microscope and counted with Image J software.

### Matrigel invasion assay

A Transwell system with 8-μm chambers (Corning, USA) in a 24-well plate was precoated with Matrigel matrix (Corning, USA) to evaluate the invasive ability of HTR-8 cells. CCR1^-^ and CCR1^+^ dMφ were pretreated with or without 100 ng/ml rhCCL8. Then, HTR-8 cells resuspended in serum-free medium were placed into the upper chamber and the lower chamber was filled with complete medium, CCR1^-^ dMφ, or CCR1^+^ dMφ. The plates were incubated at 37°C for 48 h. The inserts were washed with PBS, and non-invading cells were removed from the upper chambers. The inserts were then fixed in 4% paraformaldehyde and stained with crystal violet. The invaded cells were imaged by microscopy and quantified by counting cells in five random fields using ImageJ software.

### Single-cell transcriptomics analysis

Single-cell sequencing data were downloaded from the Gene Expression Omnibus (GEO, https://www.ncbi.nlm.nih.gov/geo/), Genome Sequence Archive (GSA) database, and ArrayExpress from EMBL-EBI. The original source of single-cell raw data is CRA002181 (32), which comes from the decidua of 15 healthy controls and 9 patients with URPL in the GSA database. The expression profile of the blood and normal decidua of early pregnancy was derived from E-MTAB-6678 (33) in the ArrayExpress database, and endometrial samples were derived from GSE111976 (34) in the GEO database.

### Data processing, high-dimensional reduction, and clustering

Fastq files of raw data were downloaded from the GSA database, and Cellranger (10X genomics) was used to process, align, and generate the feature-barcode unique molecular identifier matrices with default parameters. The gene expression matrix was analyzed using the R package Seurat (CreateSeuratObject) (35). To filter out low-quality cells, we first removed cells with detected gene numbers (<500 or >3000) and high mitochondrial content (≥10%). The expression matrix was then normalized (NormalizeData), and highly variable genes were identified by fitting the mean–variance relationship (FindVariableGenes). Next, we performed principal component analysis using highly variable genes and integrated samples using the R package Harmony (RunHarmony). The same principal components were used to embed cells in the K-nearest neighbor graph (FindNeighbors) and cluster them using the Louvain algorithm at a resolution of 0.5 (FindClusters). To label the cell clusters, we used a set of classic marker genes to annotate each cell type.

### Functional enrichment and signal pathway analyses

To investigate the function of macrophage subsets, we calculated the fold change of all detected genes (FindMarkers) and performed gene set enrichment analysis (GSEA) to determine the function of macrophage subsets in the decidua and peripheral blood using the R package fgsea.

### Cell–cell communication analysis

To infer cell–cell communication between DIC and other cell types, we used CellChat to calculate and visualize cell–cell communication.

### Statistical analysis

Statistical analysis was performed using GraphPad Prism Version 7 (GraphPad, USA). All data were first tested for normality before statistical analysis. For parametric data, Student’s t-test for two-group comparisons or one-way ANOVA for multiple group comparisons were used. For non-parametric data, Mann-Whitney test were used to compare the difference between two groups. Data are presented as mean ± SEM. The criterion for statistical significance was set at *p*< 0.05.

## Results

### Distinct CCR1^+^ dMφ in patients with URPL.

**Figure 1:**
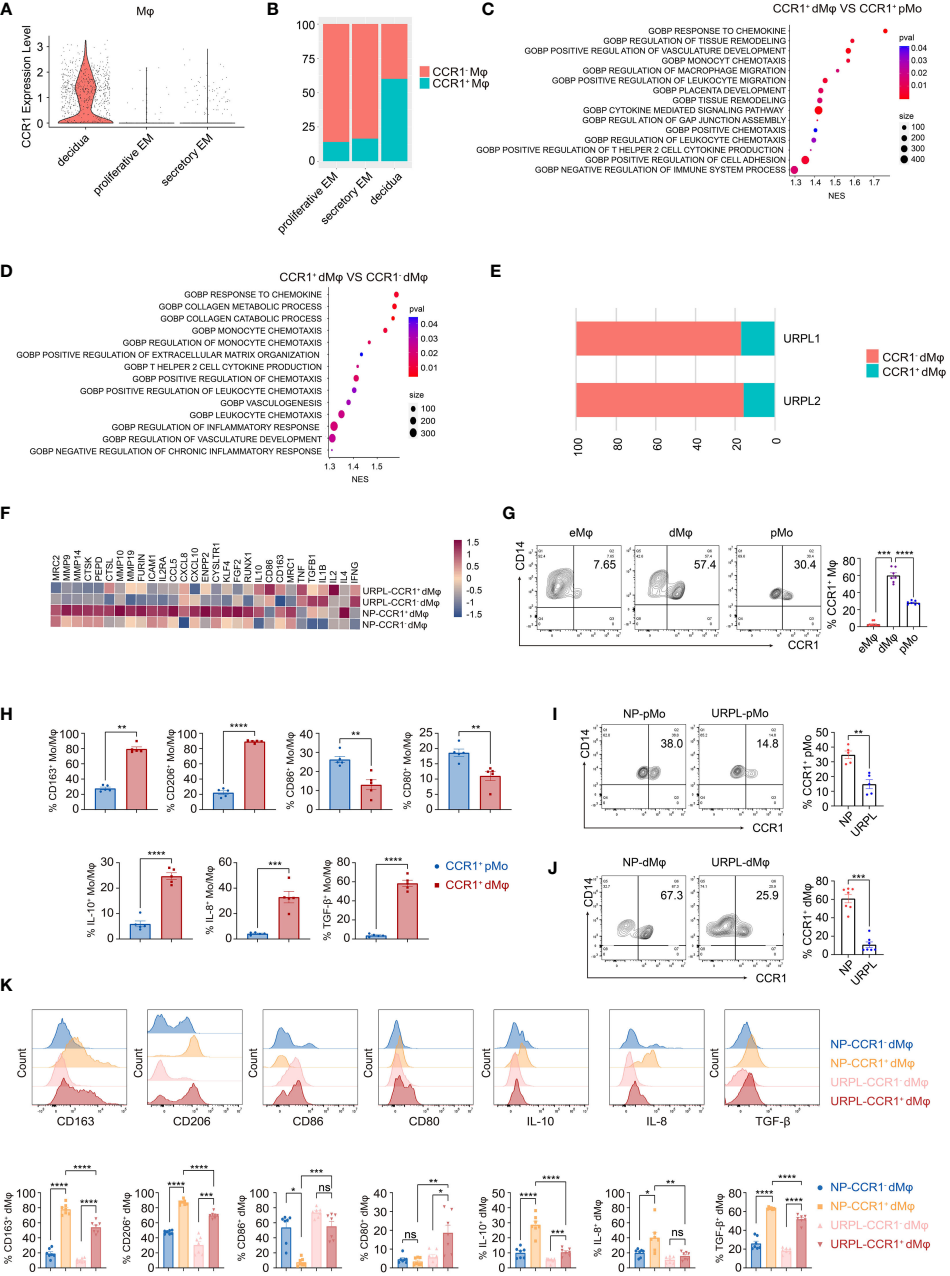
CCR1^+^ dMφ possess a distinct phenotype in URPL patients. **(A)** The violin plot representing CCR1 expression of macrophages in the endometrium and decidua. **(B)** Histogram indicating the proportion of CCR1^-^ and CCR1^+^ macrophages in the endometrium and decidua. **(C, D)** Gene Ontology (GO) analysis was conducted to identify specific pathways between CCR1^+^ peripheral monocytes (pMo) and decidual macrophages (dMφ) **(C)** and between CCR1^-^ and CCR1^+^ dMφ **(D)**. **(E)** Histogram illustrating the percentage of CCR1^-^ and CCR1^+^ dMφ in patients with unexplained recurrent pregnancy loss (URPL). **(F)** Heatmap depicting the expression of selected genes in CCR1^-^ and CCR1^+^ dMφ from normal pregnancies (NPs) and URPL. **(G)** Representative plots and quantification of CCR1^+^ eMφ (n=10), dMφ (n=7), and pMo (n=7). **(H)** FCM of the expression of indicated membrane molecules and cytokines in CCR1^+^ pMo and CCR1^+^ dMφ. 5 samples from two independent experiments. **(I, J)** Representative plots and quantification of CCR1^+^ pMo (n=5; 5) **(I)** and CCR1^+^ dMφ (n=7; 7) **(J)** between NPs and URPL. **(K)** Quantification of membrane molecules and intracellular cytokines of CCR1^-^ and CCR1^+^ dMφ from women with NPs (n=5) and URPL (n=5). Data are presented as mean ± SEM. ns, not significant; *p < 0.05, **p < 0.01, ***p < 0.001, ****p < 0.0001.

To characterize dMφ during early pregnancy, two published single-cell databases were integrated and analyzed (33, 34). The results showed that dMφ from the first trimester of pregnancy had a substantially higher expression of *CCR1* than those in the proliferative and secretory endometrium (**Figure 1A**). Based on *CCR1* expression, we divided macrophages into CCR1^+^ and CCR1^−^ subsets. A comparable percentage of CCR1^+^ Mφ was found in the proliferative and secretory endometrium; however, the infiltration of CCR1^+^ Mφ within the first trimester decidua considerably increased approximately 2–3 folds (**Figure 1B**). The decidua-specific expression of *CCR1* in macrophages suggests the influence of a unique maternal–fetal microenvironment rather than reproductive hormones. We then conducted a gene ontology (GO) analysis to decipher the functional characteristics of CCR1^+^ dMφ. Compared with CCR1^+^ peripheral monocytes (pMo) (**Figure 1C**) and CCR1^-^ dMφ (**Figure 1D**), CCR1^+^ dMφ were strongly enriched in cell migration, tissue remodeling, anti-inflammatory responses, and vascularization functions. Thus, CCR1^+^ dMφ predominantly accumulate in the uterus during early pregnancy and are characterized by an immunosuppressive propensity.

The distinct CCR1^+^ dMφ population was clearly decreased in patients with URPL (**Figure 1E**). Moreover, in CCR1^+^ dMφ from patients with URPL, genes associated with anti-inflammatory responses (e.g., *IL10*, *CD163*, *MRC1*, *TGFB1*, and *IL4*) and extracellular matrix degradation (e.g., *MMP9*, *MMP10*, *MMP14*, *MMP19*, and *ICAM1*) were less enriched, whereas those responsible for activation and pro-inflammatory responses (e.g., *CD86*, *IL1B*, *IL2*, and *TNF*) were significantly enriched (**Figure 1F**). These sequencing results suggest a distinct CCR1^+^ dMφ population with a changed proportion and functional status in patients with URPL.

To validate the results from the single-cell profiling analysis, we performed FCM and found that dMφ had a significantly higher expression of CCR1 (59.98 ± 3.13) than did maternal pMo (28.04 ± 0.81) and nonpregnant endometrial Mφ (eMφ; 2.87 ± 0.79; **Figure 1G**). Moreover, CCR1^+^ dMφ represented a more immunosuppressive phenotype with a higher expression of CD163, CD206, IL-10, IL-8, and TGF-β but lower expression of CD80 and CD86 than CCR1^+^ pMo (**Figure 1H**). In patients with URPL, remarkably decreased CCR1 expression was detected in both dMφ and pMo (**Figures 1I, J**). More importantly, the immunosuppressive phenotype of CCR1^+^ dMφ was remarkably attenuated in patients with URPL. In those with NP, CCR1^+^ dMφ exhibited a higher expression of CD163 and CD206 but lower expression of CD86 than CCR1^-^ dMφ. In patients with URPL, CCR1^+^ dMφ decreased the expression of CD163 and CD206 but increased the expression of CD80 and CD86, compared with CCR1^+^ dMφ from control donors with NPs (**Figure 1K**). The production of anti-inflammatory cytokines IL-10 and TGF-β and a proangiogenic factor, IL-8, was substantially upregulated in CCR1^+^ dMφ than in CCR1^-^ dMφ of normal controls but markedly reduced in CCR1^+^ dMφ of patients with URPL (**Figure 1K**). Collectively, these data demonstrate that CCR1^+^ dMφ exhibit an immunosuppressive and anti-inflammatory phenotype that is conducive to an immune-tolerant microenvironment, whereas decreased numbers of CCR1^+^ dMφ with dysfunction may be involved in the occurrence of URPL.

### CCR1^+^ dMφ promote epithelial-to-mesenchymal transition (EMT) to facilitate trophoblast migration and invasion

**Figure 2:**
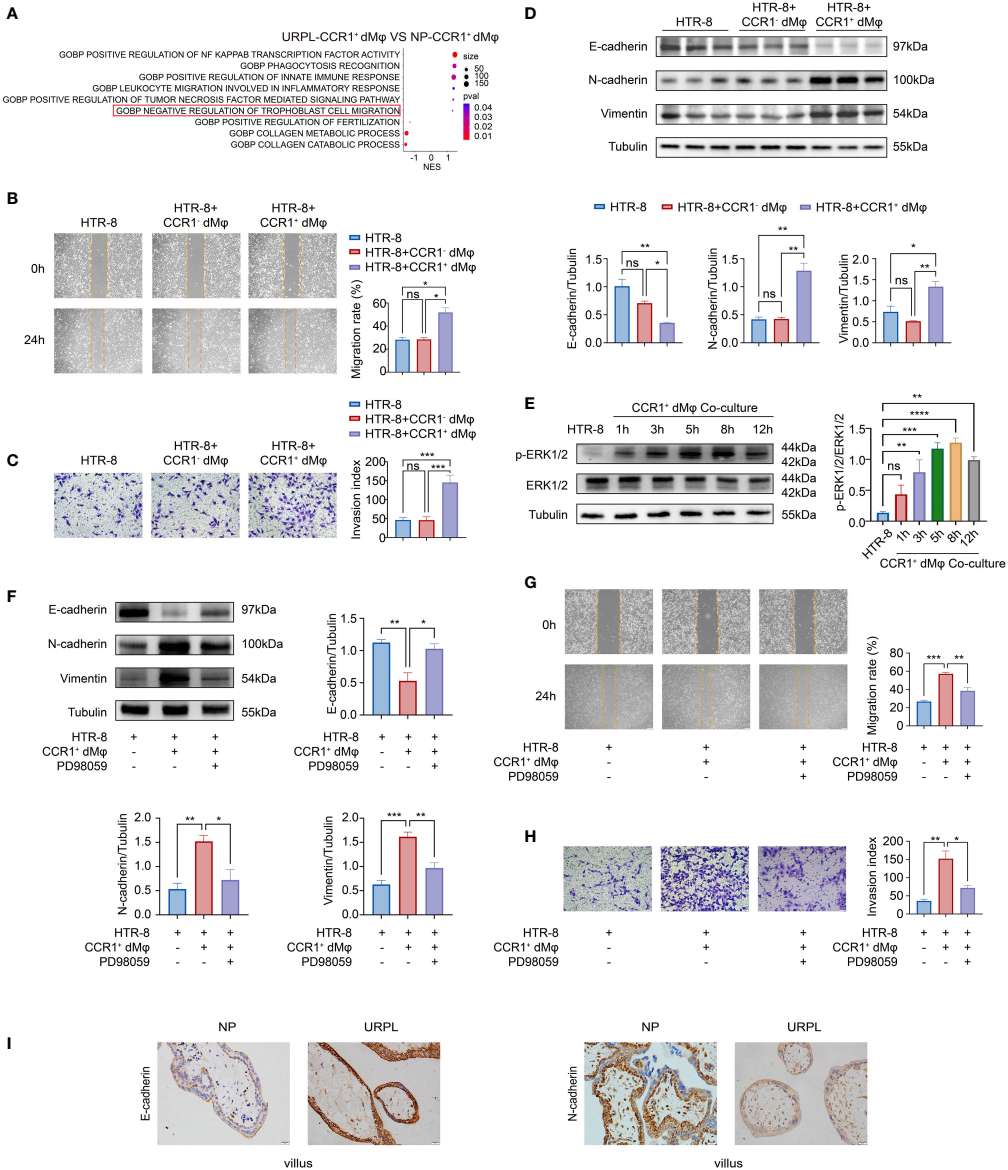
CCR1^+^ dMφ promote the migration, invasion, and EMT process of trophoblast cells. **(A)** Selected GO pathways of the DEGs of CCR1^+^ dMφ in NPs and URPL. **(B–D)** HTR-8 cells were co-cultured with CCR1^-^ dMφ or CCR1^+^ dMφ. **(B)** Scratch wound healing assay and quantitation were established to determine the migratory properties of trophoblast cells. 5 samples from two independent experiments. **(C)** Matrigel invasion assay and quantitation were performed to analyze the invasive capacity of trophoblast cells. 5 samples from two independent experiments. **(D)** Whole lysates of trophoblast cells were detected for the expression of EMT markers (n=3/group, 2 repeated experiments). **(E)** WB analysis showing the alternation of ERK1/2 pathways in HTR-8 cells co-cultured with CCR1^+^ dMφ. Images are representative of 3 samples from 3 independent experiments. **(F–H)** HTR-8 cells were pretreated with an ERK1/2 inhibitor (PD98059) before co-culture with CCR1^+^ dMφ. **(F)** Immunoblots showing the expression of E-cadherin, N-cadherin, and vimentin in HTR-8 cells treated as indicated. Images are representative of 3 samples from 3 independent experiments. **(G)** Scratch wound healing assay and **(H)** invasion assay were performed in HTR-8 cells treated as indicated. 5 samples from two independent experiments. **(I)** Representative immunohistochemical staining images showing the expression of EMT markers in human villous tissues from women with NPs (n=3) and URPL (n=3). The results were representative of three separate experiments. Data are presented as mean ± SEM. ns, not significant; *p < 0.05, **p < 0.01, ***p < 0.001.

Besides the enhanced activated and inflammatory phenotype of CCR1^+^ dMφ in patients with URPL, CCR1^+^ dMφ in these patients have a decreased function in tissue remodeling and negative regulation on trophoblast cell migration, as revealed by single-cell sequencing analysis (**Figure 2A**). Next, we probed the effects of CCR1^+^ dMφ on the biological behavior of trophoblasts. CCR1^-^ dMφ and CCR1^+^ dMφ were isolated and co-cultured with HTR-8 cells (a human extravillous trophoblast cell line) respectively in a non-contact Transwell system. Compared to co-culture with CCR1^-^ dMφ, the migration and invasion of HTR-8 cells significantly increased when co-cultured with CCR1^+^ dMφ (**Figures 2B, C**). A recent study demonstrated that EMT plays an important role in the regulation of trophoblast migration and invasion, and this process is promoted by M2 macrophages but inhibited by M1 macrophages (7). WB was performed to analyze EMT markers and signaling pathways involved in HTR-8 after co-culture with CCR1^+^ dMφ or CCR1^-^ dMφ. In the CCR1^+^ dMφ co-culture group, the expression of the epithelial marker E-cadherin was markedly decreased, whereas that of the mesenchymal markers N-cadherin and vimentin was dramatically increased in HTR-8 cells (**Figure 2D**). The expression levels of E-cadherin, N-cadherin and vimentin were comparable between the HTR-8 cells cultured alone and those co-cultured with CCR1^-^ dMφ (**Figure 2D**). Moreover, CCR1^+^ dMφ co-culture increased the phosphorylation of ERK1/2 in HTR-8 cells in a time-dependent manner (**Figure 2E**). A specific EKR1/2 inhibitor, PD98059, was applied to the CCR1^+^ dMφ-HTR-8 co-culture system and effectively abrogated the CCR1^+^ dMφ-induced EMT of trophoblasts by reducing the expression of N-cadherin and vimentin but increasing the expression of E-cadherin (**Figure 2F**). Furthermore, the migratory and invasive activities of HTR-8 cells were determined following treatment with the EKR1/2 inhibitor. As shown in **Figures 2G, H**, ERK1/2 inhibition attenuated the migration and invasion of CCR1^+^ dMφ cells. These data suggest that CCR1^+^ dMφ induce EMT to promote trophoblast migration and invasion by activating the ERK1/2 pathway. Consistent with the decreased and dysfunctional CCR1^+^ dMφ in patients with URPL, deficient EMT of trophoblasts was observed in patients with URPL, which was manifested as upregulated expression of E-cadherin and downregulated expression of N-cadherin in placental villous tissues, mainly in cytotrophoblasts (**Figure 2I**). Together, our findings suggest that dysregulated CCR1^+^ dMφ affect EMT, leading to inadequate trophoblast migration and invasion, which is involved in the occurrence of URPL.

### Upregulated CCL8 in DSCs is a candidate regulator of CCR1^+^ dMφ

**Figure 3:**
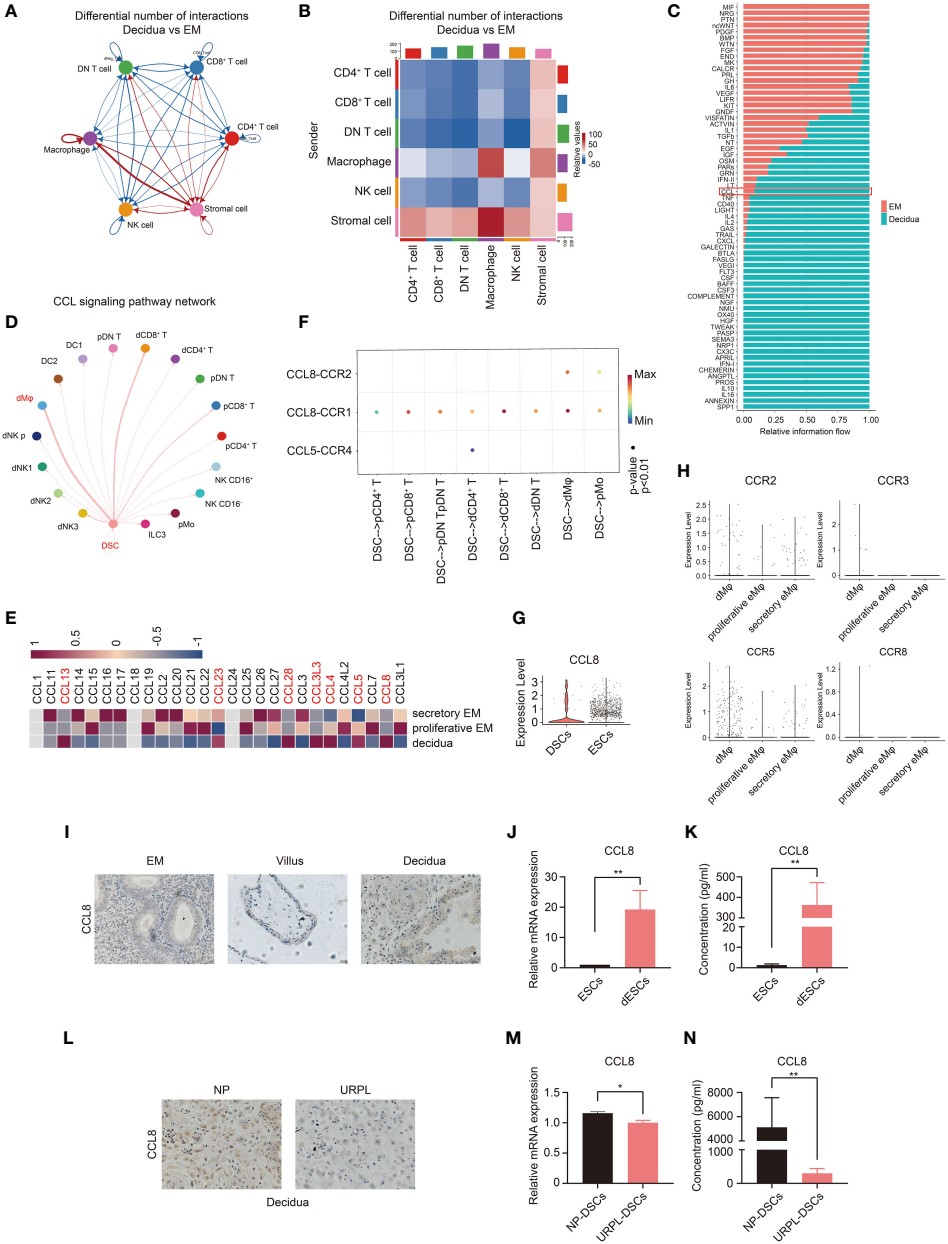
Single-cell analysis predicts the CCL8–CCR1 interaction between DSCs and macrophages during early pregnancy. **(A, B)** Number of ligand–receptor pairs between any pair of two cell populations. The edge width and shade are proportional to the indicated number of ligand–receptor pairs. **(C)** Signaling pathways were ranked based on their differences of overall information flow within the inferred networks between the endometrium and decidua. **(D)** Inferred CCL signaling networks in the decidua (circle plot). **(E)** Heatmap showing the expression of selected CCL genes in the proliferative and secretory endometrium and decidua. **(F)** The significant ligand–receptor pairs of the CCL pathway that contributed to the signaling sending from decidual stromal cells (DSCs) to immune cells. The dot color and size illustrate the calculated communication probability and p-values. **(G)** The violin plot referring to log-normalized expression values of CCL8 in DSCs and endometrial stromal cells (ESCs). **(H)** The violin plot representing log-normalized expression values of CCL8 receptors in the endometrium and decidua. **(I)** Paraffin sections of the endometrium (n=3), villous tissue (n=3), and decidua (n=3) were tested for CCL8 expression using IHC. **(J, K)** The mRNA level (n=6) and CCL8 concentration (n=10) before and after in vitro decidualization of ESCs. **(L)** Representative IHC images of CCL8 in human decidua tissues from women with NPs (n=3) and URPL (n=3). **(M, N)** The mRNA level and CCL8 concentration between DSCs from women with NPs (n=6) and URPL (n=6). Data are presented as mean ± SEM. and are representative of at least two separate experiments. *p < 0.05, **p < 0.01.

Next, we examined the possible regulator(s) of CCR1^+^ dMφ and underlying mechanisms in early pregnancy. We investigated the interaction between dMφ and other decidual cells using single-cell sequencing analysis (33, 34). CellChat analysis of single-cell sequencing in the endometrium and decidua revealed a potentially increased interaction between the stromal cells and macrophages in early pregnancy compared with that in the endometrium (**Figures 3A, B**). We also compared the overall communication probabilities of the two databases (33, 34). Intriguingly, 43 out of 66 pathways were highly active, albeit at different levels, in the decidual tissues (**Figure 3C**). The CCL signaling pathway was activated in the decidua (**Figure 3C**). As depicted in **Figure 3D**, the CCL pathway exhibited abundant signaling interactions between DSCs and dMφ. We then analyzed changes in the expression of CCL molecules and found that the expression levels of *CCL13*, *CCL23*, *CCL28*, *CCL3L3*, *CCL4*, *CCL5*, and *CCL8* were markedly higher in the decidua than in the proliferative and secretory endometrium (**Figure 3E**). Specifically, only two CCL pathways, CCL8-CCR1 and CCL8-CCR2, were significantly enriched in DSCs and dMφ (**Figure 3F**). In addition, single-cell sequencing suggested that CCL8, a ligand of CCR1, was highly expressed in DSCs compared with that in nonpregnant ESCs (**Figures 3E, G**). CCL8 can bind to several receptors, including CCR1, CCR2, CCR3, CCR5, and CCR8 (24). However, except for CCR1, other receptors were scarcely expressed on macrophages from either the nonpregnant endometrium or pregnant decidua (**Figure 3H**). Thus, decidua-derived CCL8 is a possible ligand that interacts with CCR1^+^ dMφ.

We then measured CCL8 expression at the maternal–fetal interface. As shown in **Figure 3I**, compared with the endometrium and villous, higher CCL8 expression was detected in the decidua from normal early pregnancy. Furthermore, both mRNA and protein expression levels of CCL8 were dramatically increased during *in vitro* decidualization (**Figures 3J, K**). However, immunochemistry staining showed that the decidua from patients with URPL exhibited markedly downregulated CCL8 expression compared with that from NPs (**Figure 3L**). We also identified significantly lower levels of CCL8 in DSCs from patients with URPL (**Figures 3M, N**). These findings indicate that elevated CCL8 during stromal cell decidualization is a possible regulator of CCR1^+^ dMφ, and abnormal CCL8-CCR1^+^ dMφ communication may be implicated in URPL.

### CCL8 recruits and educates CCR1^+^ pMφ into CCR1^+^ dMφ-like immunosuppressive subsets

**Figure 4:**
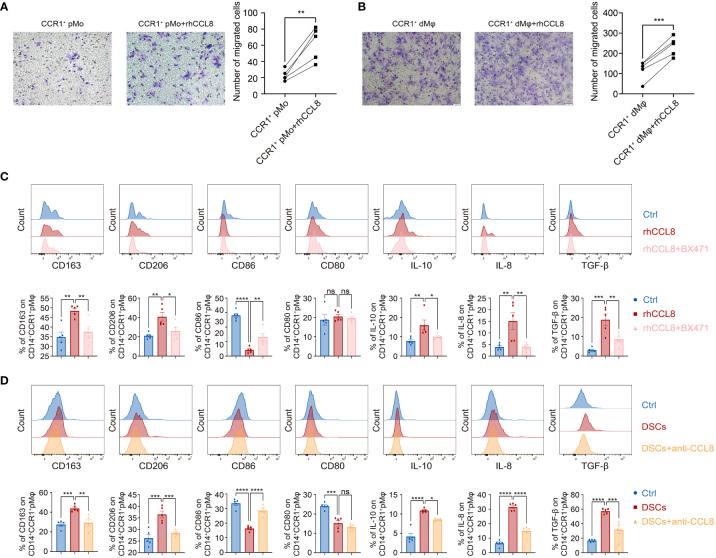
DSC-derived CCL8 recruits and educates CCR1^+^ pMφ towards CCR1^+^ dMφ phenotype. **(A, B)** Representative images and quantification of migration of the CCR1^+^ pMo (n=5 per group) **(A)** and dMφ (n=5 per group) **(B)** stimulated by rhCCL8. **(C)** Representative plots and quantification of the membrane molecules and cytokines in CCR1^+^ pMφ treated with rhCCL8 in the presence or absence of the CCR1 inhibitor, BX471 (n=5 per group). **(D)** Flow cytometric analysis and quantification of the indicated membrane molecules and cytokines in CCR1^+^ pMφ co-cultured with DSCs in the presence or absence of the CCL8 neutralizing antibody (n=5 per group). Data are presented as mean ± SEM. and are representative of at least two separate experiments. ns, not significant; *p < 0.05, **p < 0.01, ***p < 0.001, ****p < 0.0001.

Next, we investigated the regulatory effects of DSC-derived CCL8 on CCR1^+^ dMφ. We first performed a chemotaxis assay and found that CCL8, as a chemokine, potently promoted the migration of both CCR1^+^ pMo and CCR1^+^ dMφ (**Figures 4A, B**), suggesting that CCL8 contributes to the recruitment and residence of CCR1^+^ dMφ in early pregnancy.

CCR1^+^ dMφ was characterized by an anti-inflammatory phenotype in women with NP, which changed to an activated and less immunosuppressive status in patients with URPL. Whether CCL8 influences the unique phenotype of CCR1^+^ dMφ during early pregnancy remains unknown. Accordingly, pMφ were treated with recombinant human CCL8 (rhCCL8), and phenotypic changes were analyzed using FCM. Higher expression of CD163 and CD206 and lower expression of CD86 were detected in CCR1^+^ pMφ treated with rhCCL8, whereas the expression of CD80 remained unchanged. In addition, CCL8-treated CCR1^+^ pMφ produced more anti-inflammatory cytokines, including IL-10 and TGF-β, and the proangiogenic factor IL-8 (**Figure 4C**). Significantly, a specific CCR1 inhibitor, BX471, was applied during the *in vitro* stimulation of pMφ with CCL8, and all CCL8-mediated effects on pMφ, including increased expression of immunosuppressive markers and anti-inflammatory cytokines, could be abrogated by BX471. These results suggest that the CCL8–CCR1 interaction is conducive to the inactive and anti-inflammatory phenotype of macrophages at the maternal–fetal interface.

Similar results were obtained in the indirect contact co-culture system of pMφ and DSCs using Transwell chambers. DSCs effectively shifted pMφ into an anti-inflammatory phenotype with a higher expression of CD163, CD206, IL-10, TGF-β, and IL-8 but lower expression of CD80 and CD86 (**Figure 4D**). This effect was partially blocked by a neutralizing antibody against CCL8 (**Figure 4D**). Collectively, DSC-derived CCL8 can recruit CCR1^+^ pMo from the maternal periphery and induce an immunosuppressive phenotype, contributing to maternal–fetal immune tolerance.

### CCL8 enhances the function of CCR1^+^ dMφ in promoting trophoblast migration and invasion

**Figure 5:**
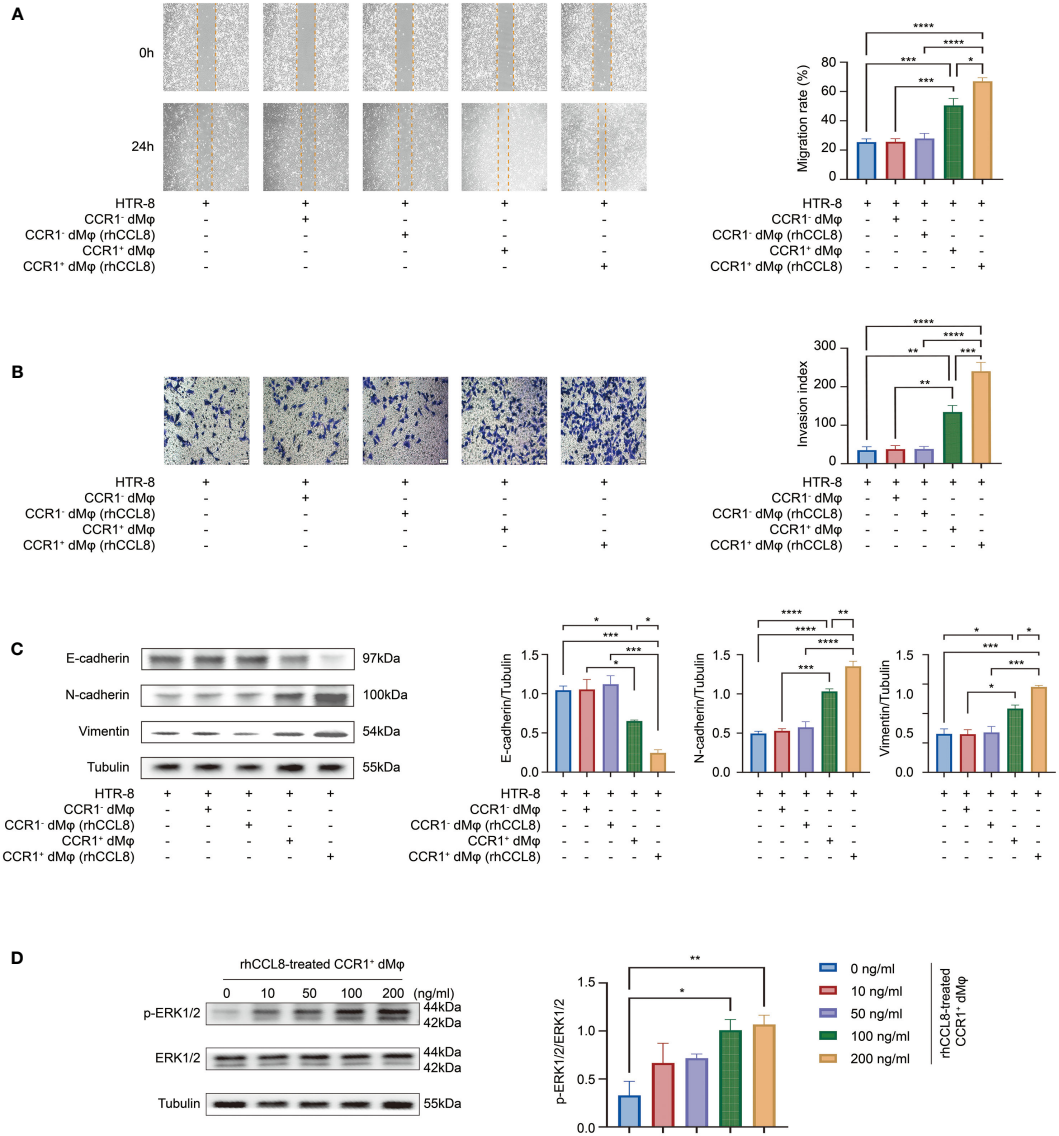
CCL8 enhances the effect of CCR1^+^ dMφ on trophoblast functions. **(A–C)** HTR-8 cells were co-cultured with CCR1^-^ dMφ or CCR1^+^ dMφ pretreated with or without rhCCL8. **(A)** Scratch wound healing assay and quantitation were used to represent trophoblast migration (n=5 per group, 2 independent experiments). **(B)** Invasion assay and quantitation were applied to show the invasive activity of trophoblast cells (n=5 per group, 2 independent experiments). **(C)** WB was performed to examine the expression of E-cadherin, N-cadherin, and vimentin in HTR-8 cells treated as indicated. Images are representative of 3 samples from 3 independent experiments. **(D)** Effects of CCR1^+^ dMφ pretreated with different dosages of rhCCL8 on the activation of the ERK1/2 pathway in HTR-8 cells. Images are representative of 3 samples from 3 independent experiments. Data are presented as mean ± SEM. *p < 0.05, **p < 0.01, ***p < 0.001, ****p < 0.0001.

We have demonstrated that CCR1^+^ dMφ could promote the migration, invasion, and EMT of trophoblast cells. Whether CCL8 has an impact on this function of CCR1^+^ dMφ is unknown. Therefore, HTR-8 cells were co-cultured with rhCCL8 preteated-CCR1^+^ dMφ or CCR1^-^ dMφ, followed by migration and Transwell assays. Consistent with the above results, CCR1^+^ dMφ significantly facilitated the functions of HTR-8 cells, while CCR1^-^ dMφ, whether treated with rhCCL8 or not, had less effects on migration, invasion or EMT process of HTR-8 cells (**Figures 5A–C**). Compared with CCR1^+^ dMφ, CCL8-pretreated CCR1^+^ dMφ showed a significantly enhanced capacity to promote trophoblast migration and invasion (**Figures 5A, B**). Moreover, CCL8-pretreated CCR1^+^ dMφ co-culture further increased the expression of N-cadherin and vimentin and decreased the expression of E-cadherin in trophoblast cells (**Figure 5C**), indicating a more activated EMT process than that induced by CCR1^+^ dMφ. In addition, CCR1^+^ dMφ pretreated with different concentrations of CCL8 promoted the activation of the ERK1/2 pathway in trophoblast cells in a concentration-dependent manner (**Figure 5D**). These data suggest that CCL8 has a positive effect on the function of CCR1^+^ dMφ in promoting EMT-mediated trophoblast migration and invasion.

## Discussion

**Figure 6:**
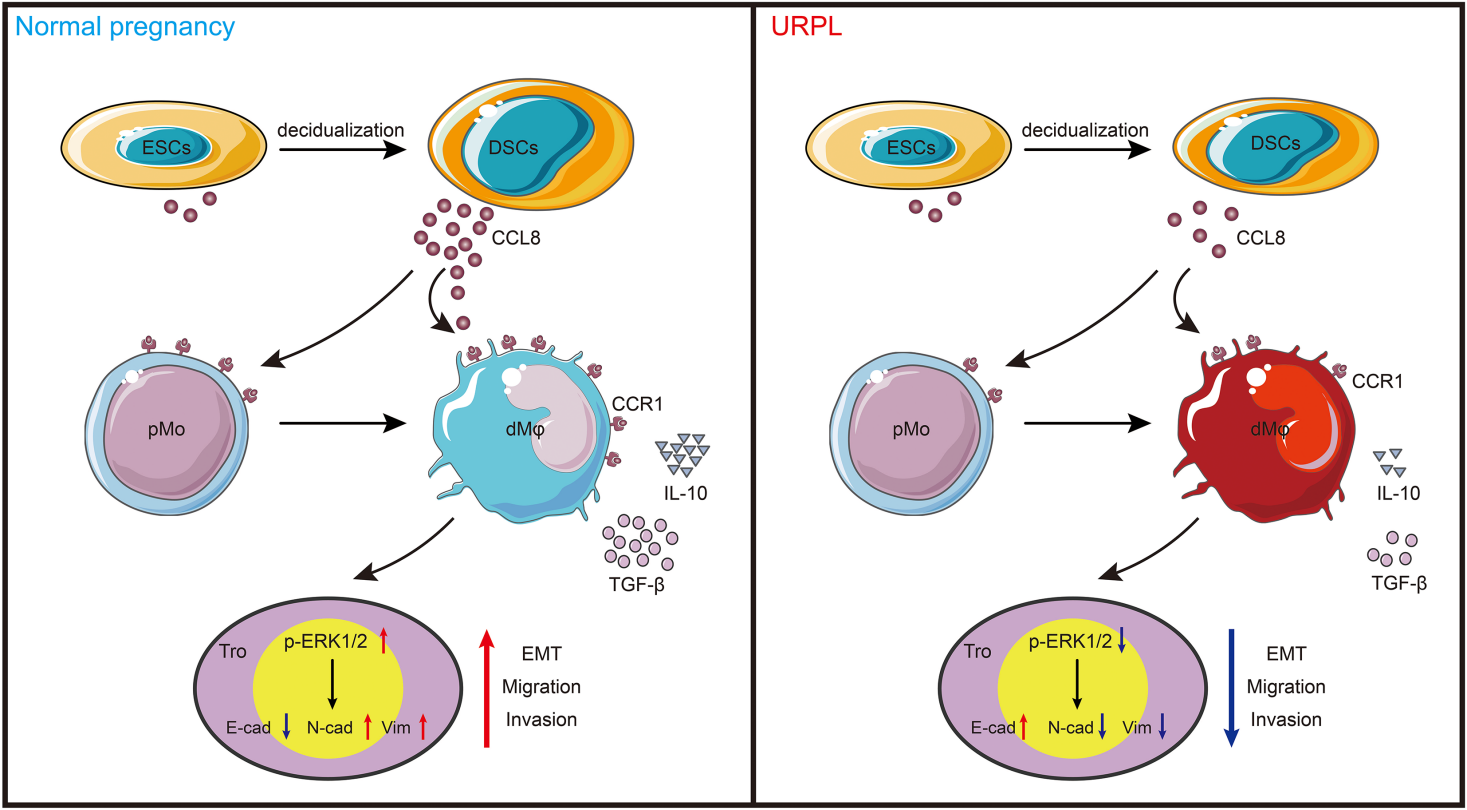
Schema of how CCR1^+^ dMφ function at the maternal–fetal interface. CCL8 expression is increased substantially during endometrial decidualization. Through interaction with CCR1, CCL8 attractes CCR1^+^ pMo from peripheral blood and skews them into a CCR1^+^ dMφ-like phenotype, which displays immunosuppressive phenotype and produces more anti-inflammatory cytokines. This crosstalk further supports the migratory and invasive capacities of trophoblasts during EMT by activating the ERK1/2 pathway. Conversely, poor decidualization, characterized by reduced CCL8 secretion, results in decreased number of and dysfunctional CCR1^+^ dMφ, which impair the normal functions of trophoblast cells in women with URPL.

This study is the first one to report dMφ-specific expression of CCR1 in early pregnancy. Based on CCR1 expression, dMφ can be divided into two distinct subsets. Among them, CCR1^+^ dMφ possessed an anti-inflammatory signature and was able to promote the migration, invasion, and EMT process of trophoblast cells through the ERK1/2 signaling pathway. Moreover, DSC-derived CCL8 was identified as a regulator of CCR1^+^ dMφ. Through its interaction with CCR1, CCL8 recruited CCR1^+^ monocytes from the maternal periphery and further instructed these cells into a CCR1^+^ dMφ-like phenotype. The CCR1^+^ dMφ-mediated function of trophoblasts was also strongly reinforced by CCL8. More importantly, the crosstalk among DSCs, CCR1^+^ dMφ, and trophoblasts was weakened in patients with URPL, which manifested as decreased CCL8 expression in DSCs and abolished proportion of CCR1^+^ dMφ, accompanied by a pro-inflammatory phenotypic transformation and deficient EMT of trophoblasts. Our results indicate that CCR1^+^ dMφ play an important role in immune tolerance and trophoblast functions at the maternal–fetal interface. Dysfunctional CCR1^+^ dMφ are closely associated with the defective crosstalk between DSCs and trophoblast cells, which is implicated in the pathogenesis of URPL (**Figure 6**).

Abnormal numbers and proportions of macrophages have been observed in early pregnancy loss, although the current results are controversial and lack a specific mechanism. Yang et al. found fewer dMφ in patients with spontaneous abortion and aborted mice compared with that in women with NPs and normal pregnant mice. One study applied IHC and reported no significant differences in the ratio of dMφ to DICs in patients with URPL (36). Recently, with the rapid development of the single-cell sequencing technology, the composition and phenotype of immune cells at the maternal–fetal interface have been further understood. Chen et al. found that dMφ showed a slightly increased ratio in patients with URPL compared with that in normal tissues (37). However, validated by both single-cell sequencing analysis and FCM, women with URPL showed a decreased dMφ population (32). In our study, we found significant enrichment of CCR1^+^ Mφ (around 60%) in the decidua during early pregnancy and a small proportion (<10%) of this subset in the nonpregnant endometrium. However, in patients with URPL, the percentage of CCR1^+^ dMφ was substantially reduced, suggesting that decreased total dMφ in URPL was mainly due to the decrease in the CCR1^+^ dMφ subset. The origin of dMφ includes embryonic origin and recruitment of peripheral monocytes (38). dMφ are recruited and enriched by VEGF, CSF-1, and RANTES (39, 40) and can adhere and reside in the decidua under the action of CD74, RANKL, and lysophosphatidic acid (4, 41, 42). Here, we identified that CCL8, which was upregulated in DSCs during the first trimester, displayed an efficient chemotactic effect on both CCR1^+^ pMo and CCR1^+^ dMφ. Interestingly, the expression of DSC-derived CCL8 and proportion of CCR1^+^ pMo were remarkably downregulated in patients with URPL. Thus, attenuated CCL8-mediated recruitment and residence of CCR1^+^ dMφ may contribute to the decreased total dMφ in patients with URPL.

Over the years, macrophages have been categorized under the conventional M1/M2 classification, and dMφ are no exception. dMφ possess a variety of M2-type macrophage surface molecules, such as CD206 and CD163, and highly express anti-inflammatory factors; thus, dMφ are widely accepted as M2 in NP (11). Activation of dMφ toward the M1 phenotype and increased number of M1 dMφ have been correlated with the pathology of URPL (43, 44). However, with deep single-cell analysis, multiple studies have found that the expression profiles of macrophages in different tissues display significant diversity (45–47). Here, we identified CCR1^+^ and CCR1^-^ dMφ in the decidua from early pregnancy. Previous reports have shown that CCR1 expression was the lowest in M1 macrophages but the highest in tumor-educated macrophages (48). Similarly, compared with CCR1^-^ dMφ, CCR1^+^ dMφ in early pregnancy exhibited a significant anti-inflammatory phenotype, characterized by a high expression of CD163 and CD206 and increased production of IL-10 and TGF-β. These results indicated an M2-like phenotype in CCR1^+^ dMφ and an M1-like phenotype in CCR1^-^ dMφ; thus, dMφ as a whole maintain immunosuppressive properties as well as defense capability. However, in women with URPL, the frequency of CCR1^+^ dMφ was sharply decreased and the immunosuppressive status of these cells was changed to hyperactivated and inflammatory, leading to imbalanced maternal–fetal immune responses unbeneficial to pregnancy maintenance.

The function of CCR1^+^ dMφ was also investigated in this study, which demonstrated that CCR1^+^ dMφ promote trophoblast migration and invasion by inducing the EMT process *via* the activation of the ERK1/2 signaling pathway. This function of CCR1^+^ dMφ was enhanced by CCL8. However, we detected deficient EMT in trophoblasts, reduced CCL8 expression in DSCs, and decreased number and dysfunction of CCR1^+^ dMφ in URPL. Emerging evidence has shown that proper and sufficient trophoblast migration and invasion are essential for embryo implantation, placental formation, and spiral artery remodeling (49, 50). The EMT process refers to a cellular change in which the polarity and cell adhesion properties decrease and cell migration and invasion increase, which is accompanied by trophoblast invasion (51, 52). Inadequate and defective EMT process in trophoblast cells is an etiological factor associated with pregnancy complications, including URPL, preeclampsia, and intrauterine growth retardation (53, 54). The initiation and maintenance of trophoblast EMT are greatly influenced by the maternal–fetal microenvironment, including local immune cells. Through the production of IL-8 and IP-10, decidual natural killer cells can stimulate the recruitment and migration of trophoblast cells (55). Studies have found that activated M1 macrophages induced by lipopolysaccharide dampen trophoblast migration and invasion by producing high levels of TNF-α through the regulation of the E-cadherin/β-catenin pathway (16, 56). IL-10 derived from M2 macrophages can reverse the effect of TNF-α-induced poor invasive properties of trophoblast cells. Additionally, M2 macrophages can promote EMT, invasion, and migration of trophoblasts by secreting G-CSF *via* the activation of the PI3K/AKT/ERK1/2 pathway (57). Therefore, our findings suggest that dysfunction of CCR1^+^ dMφ may account for the downregulated EMT process in trophoblasts, which leads to limited trophoblast migration and invasion in patients with URPL.

Macrophages are highly plastic to the disturbance of homeostasis and are conditioned by the local tissue environment (58). CCR1 was initially identified as a receptor of CCL3 and CCL5. With the discovery of additional chemokines in CC categories, studies have revealed that CCR1 binds and functions in response to a range of chemokines, such as CCL7, CCL8, CCL14, and CCL15 (24, 59, 60). In our study, CCL8 potently modulated the origin, phenotype, and function of CCR1^+^ dMφ in early pregnancy, as demonstrated by single-cell analysis and functional experiments. CCL8 is a crucial regulator of the homing of TH2 cells and drives chronic allergic inflammation by interacting with CCR8 (61, 62). Furthermore, mainly produced by airway macrophages, CCL8 elicits the movement and activation of type 2 innate lymphoid cells (ILC2) during inflammation (63). Studies investigating the roles and mechanisms of action of CCL8 in macrophages are limited. The ablation of CCL8 in mice implanted with breast cancers reversed the chemoattractant effect of M2 macrophages in a CCR2-dependent manner (64, 65). Zhang et al. detected an increase in CCL8 expression, resulting in the enrichment of myeloid cells, restoration of immune suppression, and acceleration of carcinogenesis, which were blocked by the CCL8 receptor CCR1 (48). Our results revealed that either exogenous CCL8 or DSC-derived CCL8 could promote the transformation of CCR1^+^ pMφ into a more anti-inflammatory phenotype, which could be abrogated by the CCL8 neutralizing antibody. CCL8 improved the function of CCR1^+^ dMφ in promoting EMT, migration, and invasion of trophoblast cells. Therefore, as a potent regulator of CCR1^+^ macrophages, CCL8 is of great significance for establishing an immune-tolerant microenvironment and adequate trophoblast functions.

Collectively, our study identified a distinct dMφ subpopulation, CCR1^+^ dMφ, that exhibit immunosuppressive activity during early pregnancy. The decreased frequency of CCR1 in dMφ was accompanied by downregulated inhibitory membrane molecule expression and dysfunctional anti-inflammatory cytokine production in women with URPL. We also determined that CCR1^+^ dMφ could promote migratory and invasive traits and EMT in trophoblast cells by activating the ERK1/2 pathway. Moreover, the recruitment of CCR1^+^ dMφ, as well as their immunotolerant phenotype and regulatory functions in trophoblasts, all depend on the interaction with DSC-derived CCL8. Our findings illustrate a distinct dialogue among CCR1^+^ dMφ, trophoblast cells, and DSCs during early pregnancy and underscore the critical role of CCR1^+^ dMφ subsets in URPL. These data provide insight into the immune mechanisms of URPL and potential targets for intervention.

## Data availability statement

The original contributions presented in the study are included in the article/supplementary material. Further inquiries can be directed to the corresponding authors.

## Ethics statement

The studies involving human participants were reviewed and approved by Obstetrics and Gynecology Hospital, Fudan University. The patients/participants provided their written informed consent to participate in this study.

## Author contributions

YS performed the experiments, analyzed data, and drafted the first version of manuscript. YL designed experiments, researched literatures and edited the manuscript. LX helped to carry out the experiments. JC assisted with data analysis. DL and MD conceived the project, supported the research and revised the manuscript. All authors contributed to the article and approved the submitted version.

## Funding

This work was supported by the National Basic Research Program of China (2021YFE0206500, 2017YFC1001403), National Nature Science Foundation of China (31900663, 31970859, 81630036, 81501334, 91542116, 32070915, 31900663), the Innovation-Oriented Science and Technology Grant from NHC Key Laboratory of Reproduction Regulation (CX2017-2) and the international cooperation project between Macau and Shanghai (20410760300), the Strategic Collaborative Research Program of the Ferring Institute of Reproductive Medicine (FIRMA200504), the funding of Innovative research team of high-level local universities in Shanghai.

## Acknowledgments

We thank technical support provided by DL and MD and appreciated the help from members in our laboratory.

## Conflict of interest

The authors declare that the research was conducted in the absence of any commercial or financial relationships that could be construed as a potential conflict of interest.

## Publisher’s note

All claims expressed in this article are solely those of the authors and do not necessarily represent those of their affiliated organizations, or those of the publisher, the editors and the reviewers. Any product that may be evaluated in this article, or claim that may be made by its manufacturer, is not guaranteed or endorsed by the publisher.
